# LMX1A inhibits *C-Myc* expression through ANGPTL4 to exert tumor suppressive role in gastric cancer

**DOI:** 10.1371/journal.pone.0221640

**Published:** 2019-09-26

**Authors:** Peiyu Qian, Jian Li, Xiaohong Zhang, Fan Li, Songhua Bei, Huanqing Li, Qi Sun, Li Feng

**Affiliations:** 1 Minhang Fudan Medical Research Cooperative Development Research Institute, Minhang Hospital, Fudan University, Shanghai, Minhang District, Shanghai, P.R. China; 2 Endoscopy Center, Minhang Hospital, Fudan University, Shanghai, Minhang District, Shanghai, P.R. China; The University of Hong Kong, HONG KONG

## Abstract

Our research group has showed that the LIM homeobox transcription factor 1 alpha (LMX1A) is inactivated in gastric cancers. Overexpression of LMX1A inhibits tumor growth. However, the mechanisms remains unclear. Considering LMX1A as a transcription factor, a comparison of RNA-seq between gastric cancer cells (GCCs) and GCCs with LMX1A overexpressed was performed to identify genes transcriptionally activated by LMX1A. Among the potential LMX1A target genes, angiopoietin-like 4 (ANGPTL4) has been reported to be an important tumor suppressor and thus was selected for further validation and research. Both LMX1A and ANGPTL4 showed downregulated expression in gastric cancer samples. More importantly, the expression of LMX1A is positively correlated with ANGPTL4, without including other family members in gastric cancer cell lines. What’s more, knockdown of ANGPTL4 rescued the tumor suppressive phenotype of LMX1A overexpression, which indicated that LMX1A upregulates ANGPTL4 to exert its role. Mechanistically, we found that LMX1A inhibited the expression of the oncogene *C-Myc*, which is alleviated by ANGPTL4 knockdown. In general, our results showed that LMX1A exerts its tumor suppressive role by activating ANGPTL4 to inhibit *C-Myc*.

## Introduction

LMX1A is a member of the group of LIM-homeobox-containing genes that encode LIM-homeodomain (LIM-HD) proteins[1]. LMX1A is initially known to participate in developmental events[2, 3]. It is proven that LMX1A is a critical regulator of cell-fate decisions using genetic fate mapping in wild-type and LMX1A deficient mice[3]. LMX1A also plays a pivotal role in the mDA differentiation of human embryonic stem (hES) cells. In the developing cerebellum, loss of LMX1A completely abolishes roof plate induction in the spinal cord[2]. Later on, evidence for the role of LMX1A in cancers has been found. LMX1A gene has been reported to be hypermethylated in its promoter region in gastric cancer[4], ovarian cancer[5] and cervical cancer[6], an important mechanism for loss of function of several tumor suppressor genes. Indeed, the role of LMX1A as a tumor suppressor has been unravelled in gastric cancer by us[4], and in ovarian cancer by others[7]. Also, LMX1A inhibits cancer metastasis-related functions[7, 8].

The evidence of LMX1A’s role as a tumor suppressor is piling up. However, little is known about the mechanisms through which it exerts its tumor suppressive role. Although LMX1A has been reported to be a transcription factor and act as a positive regulator of insulin gene transcription[9], no other LMX1A-regulated genes have been reported, especially in cancer. In this study, by identifying LMX1A-regulated genes using RNA sequencing in gastric cancer cell lines, we show that LMX1A exerts its tumor suppressive role partly through up-regulating ANGPTL4.

## Material and methods

### Cell lines and plasmid construction

The human gastric cancer cell lines AGS and MKN45 were used in our experiment. Both two cell lines were cultured in DMEM with 10% FBS and incubated at 37 °C with 5% CO_2_. The construction of pBabe-Flag-LMX1A, shANGPTL4#1 and shANGPTL4#2 and cell transduction process has referenced our previously published paper[10]. Western blot analysis was performed as described before [4].

### RNA extraction and RNA-seq

The total RNA was extracted from cultured cells by using RNeasy FFPE Kit (Qiagen, Valencia, CA). After the total amounts of RNA quantified, the depletion of rRNA and establishment of cDNA library was made by using Collibri^™^ Stranded RNA Library Prep Kit for Illumina^™^ Systems with Human/Mouse/Rat rRNA Depletion Kit (Invitrogen, Carlsbad, CA). The HMR rRNA Depletion Kit contained a variety of enzymes catalyzing specific depletion of rRNA sequences and remained the most noncoding RNA. Then the purified RNA was fragmented using RNase III, following a hybridization and ligation of the adaptors. After reverse transcription and Indexing PCR amplification, the cDNA library was ready for the following Illumina^™^ sequencing. the sequencing process and following data analysis was performed by Shanghai Biotecan Medical Diagnostics Co.,Ltd. For validation of the quality of RNA-seq data, each RNA-seq data was checked through Fast QC software, which includes the throughput/coverage/alignment quality analysis. The checked information was attached in the supporting information 1(S1 File).

### Tumorigenicity assay

A total of fifteen BALB/c nude mice were used in our experiment and the animal ethic was approved by Minhang Hospital Ethic Committee. Five mice were included in each group with a subcutaneous injection of three different transgenic AGS gastric cancer cell lines (EV-shNC, LMX1A-shNC, LMX1A-shANGPTL4#1). For tumor establishment, about 1 × 10^6^ transduced AGS cells were injected into each mouse and incubated for 1.5 months. Tumor sizes were estimated using the equation V = 4/3π × L/2×(W/2)^2^, where L is the mid-axis length and W is the mid-axis width. Each mouse was executed by carbon dioxide asphyxiation.

### Chromatin Immunoprecipitation (ChIP) assay

The cells were fixed by formaldehyde (1.5% in PBS) for 10 min at RT. Then glycine was added to 125 mM to stop the reaction, and the fixed cells was washed, lysed, and sonicated to break DNA into 200-1000bp fragments. The cross-linked chromatin/DNA complexes were incubated with antibodies to LMX1A (2ug/10^6^ cells ab139726, Abcam, Cambridge, UK) 1h at 4°C, following with addition of 60ul blocked Protein A/G Agarose Beads and incubation at 4°C overnight. The precipitated complexes were then washed, eluted, reverse-crosslinked, and treated with RNase A, proteinase K to purify the immune precipitation. After extraction of DNA, quantitative real-time PCR analysis was performed to observe the enrichment of ANGPTL4, The predicted PCR product included several LMX1A binding sites.

### Quantitative real-time PCR

Total RNA was extracted from cells using TRIZOL (Invitrogen, Carlsbad, CA). Reverse transcription was carried out using M-MLV transcriptase (Promega, Foster City, CA). Quantitative real-time PCR (q-PCR) was performed using a SYBR Green kit to detect the expressions of ANGPTL4 following manufacturer’s instruction (Applied Biosystem) with the following primers: 5’-GAGTTGCTGCAGTTCTCCGT-3’ (forward) and 5’-AAACCACCAGCCTCCAGAGA-3’ (reverse) for ANGPTL4. The pair of primer 5’-CATCCTCACCCTGAAGTACCC-3’ (forward) and 5’-AGCCTGGATAGCAAC GTACATG-3’ (reverse) for ACTIN served as internal control.

### Statistical analysis

Unless described, the p values for comparison between groups were obtained by Student’s t test. All statistical tests were two-sided, and p value <0.05 was considered to be statistically significant.

## Results

### Identification of LMX1A-regulated genes

**Fig 1:**
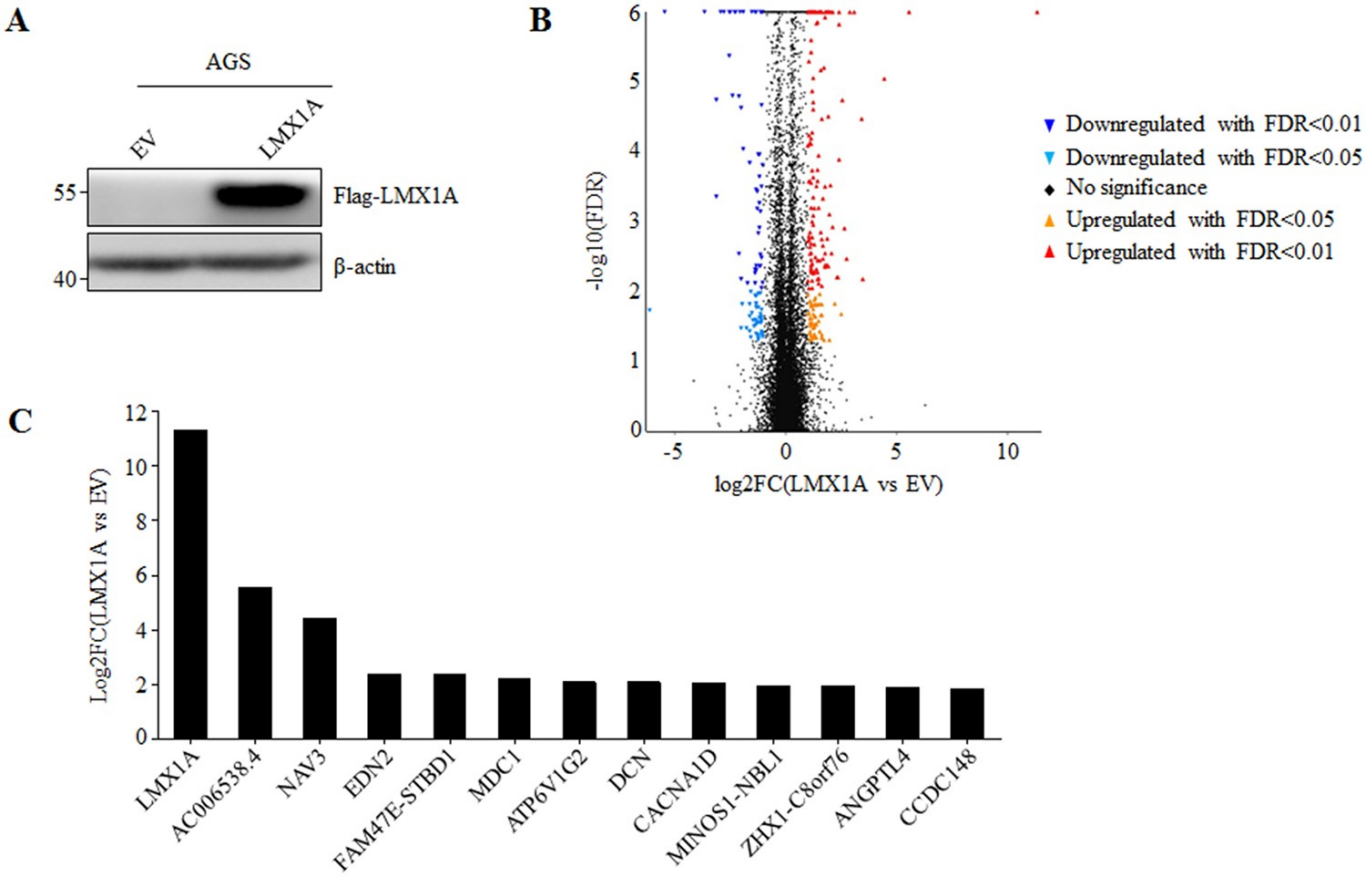
Identification of LMX1A-regulated genes by using RNA-sequencing. (A) AGS cells stably expressing empty vector (AGS-EV) or Flag-tagged LMX1A (AGS-LMX1A) were constructed by retrovirus infection. (B) AGS-EV and AGS-LMX1A cells were subjected to RNA-Seq, and volcano plot of significantly regulated gene were shown. (C) Genes that were up-regulated by LMX1A overexpression for more than 3.5 folds compared to EV-expressed cells.

**Fig 2:**
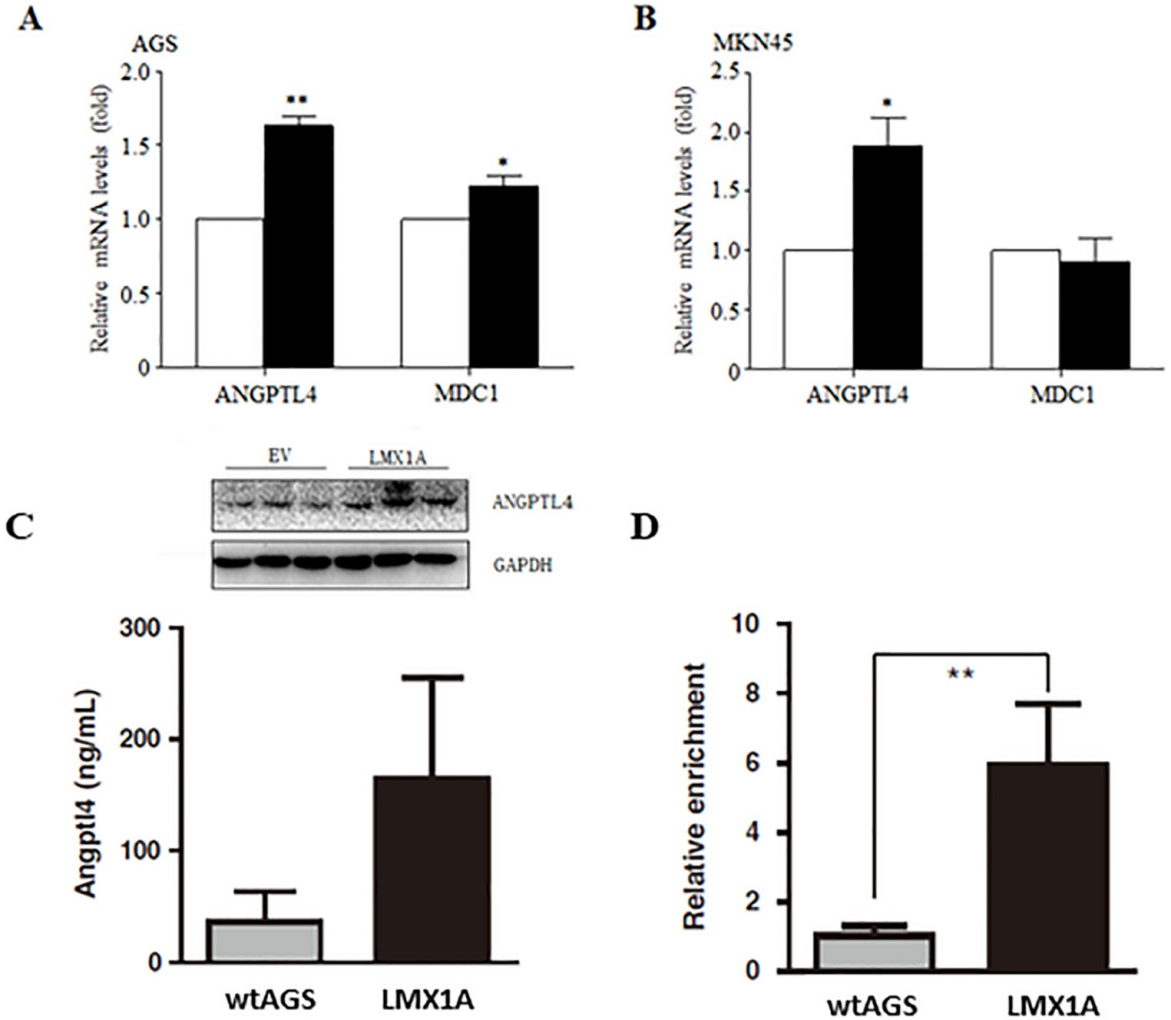
RNA-level and protein-level verification of ANGPTL4 activation by LMX1A. EV or LMX1A was overexpressed in AGS (A) and MKN45 (B) cells, and Q-PCR and western blot (C) was performed to detect the expression of ANGPTL4 and MDC1; D. ChIP assay of AGS with EV and overexpressed LMX1A, the ANGPTL4 enrichment was calculated in fold-values by normalization to the negative control IgG and input control. Student’s t test. *, P<0.05; **, P<0.01.

To identify genes that contribute to LMX1A’s tumor suppressive role, we sought to identify LMX1A-regulated genes. For this purpose, gastric cancer cell line AGS cells were infected with retrovirus carrying LMX1A-expressing plasmids, with empty vector (EV) as control. The expression of Flag-tagged LMX1A was successfully detected (Fig 1A). Total RNA was then extracted from both AGS-EV and AGS-LMX1A cells, and subjected to RNA sequencing. Fig 1B shows altered gene expression by overexpression of LMX1A compared to EV-expressed cells. To select genes most related to LMX1A, we focused on up-regulated genes and set the threshold at 3.5 folds, and 13 genes meet these criteria (Fig 1C). Among these genes, mediator of DNA damage checkpoint protein 1 (MDC1) and angiopoietin-like protein 4 (ANGPTL4) have been reported to play a tumor suppressive role in different cancer types[11, 12]. Thus, we used quantitative realtime-PCR (q-PCR) to confirm the positive regulation of LMX1A upon MDC1 and ANGPTL4 in AGS and another gastric cancer cell line MKN45 cells. The results showed that while ANGPTL4 manifested up-regulated expression in both cell lines, the up-regulated expression of MDC1 could only be confirmed in AGS cells (Fig 2A and 2B). Therefore, we chose ANGPTL4 for further analysis.

### Co-expression of LMX1A and ANGPTL4 in gastric cancer samples

**Fig 3:**
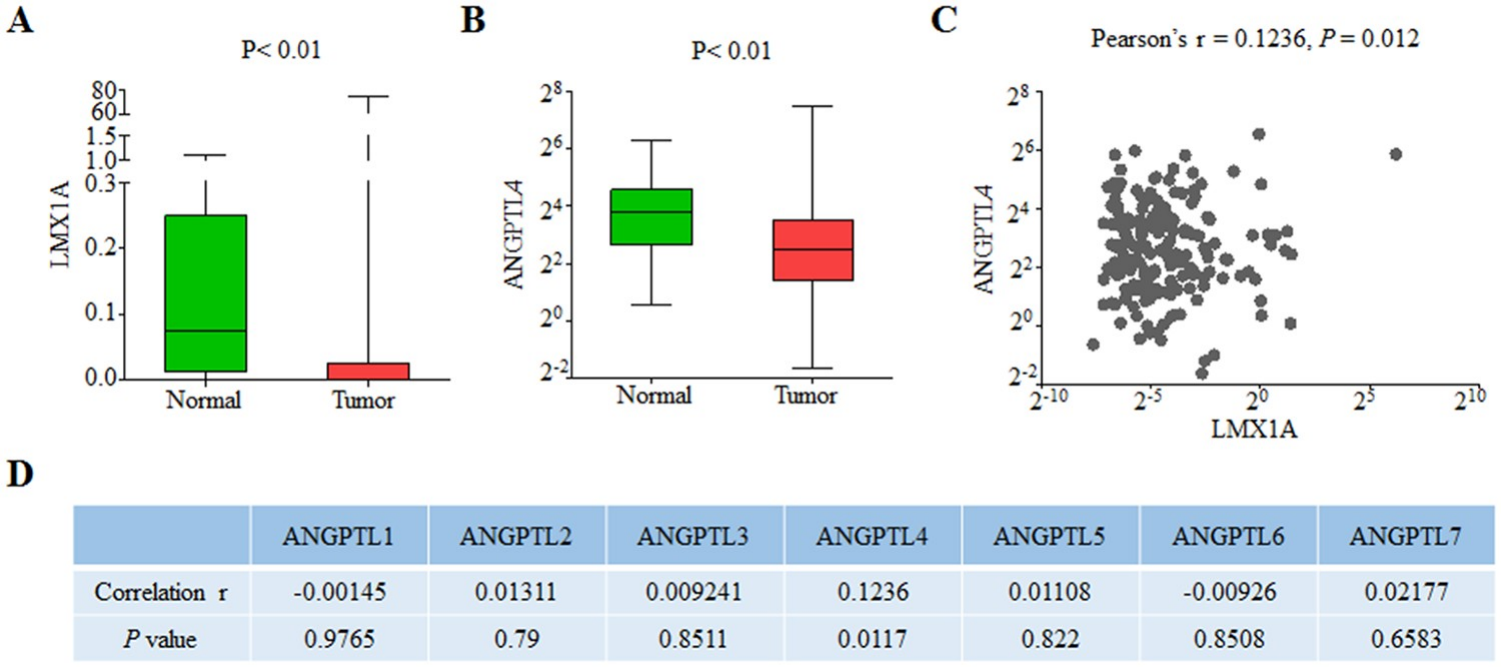
LMX1A and ANGPTL4 are downregulated and positively correlated in gastric cancer samples. (A, B) Box plots of the expression of LMX1A (A) and ANGPTL4 (B) in gastric cancer samples compared to that in normal samples in TCGA Stomach Adenocarcinoma data collection. Data were analyzed using Mann-Whitney U test. (C) Pearson’s correlation of LMX1A and ANGPTL4 in cancer samples of TCGA Stomach Adenocarcinoma data collection. (D) Pearson’s correlation of LMX1A and ANGPTL1-7 in cancer samples of TCGA Stomach Adenocarcinoma data collection.

Considering LMX1A as a transcription factor, we proposed that LMX1A could contribute to the ANGPTL4 expression and inhibit tumor growth in gastric cancer. To confirm this notion, a transcription factor database search analysis revealed that the promoter of Angptl4 includes several consensus sequences for LMX1A (S1 Table). And the western results (Fig 2C) and ChIP analysis (Fig 2D) also showed an increased enrichment of ANGPTL4 followed with an upregulation of LMX1A. Further, we investigated the relationship between LMX1A and ANGPTL4 in clinical gastric cancer samples by using The Cancer Genome Atlas (TCGA) Stomach Adenocarcinoma data collection. We confirmed that the expression of LMX1A was indeed dramatically reduced in gastric cancer samples compared to health group samples (Fig 3A), following decreased ANGPTL4 expression (Fig 3B), which suggested the positive-regulatory relationship between LMX1A and ANGPTL4 also existed in gastric cancer samples. Pearson’s correlation showed that the expressions of LMX1A and ANGPTL4 were positively correlated (Fig 3C). The correlation between LMX1A and ANGPTL4 was proved to be specific also, as LMX1A showed no correlations with other ANGPTL family members, including ANGPTL1, ANGPTL2, ANGPTL3, ANGPTL5, ANGPTL6 and ANGPTL7 (Fig 3D). Thus, our results showed an upregulation of LMX1A is related with increased ANGPTL4 expression in gastric cancer cells, which could further contribute to the gastric tumor growth inhibition.

### Knockdown of ANGPTL4 reverses the tumor suppressive role of LMX1A overexpression

**Fig 4:**
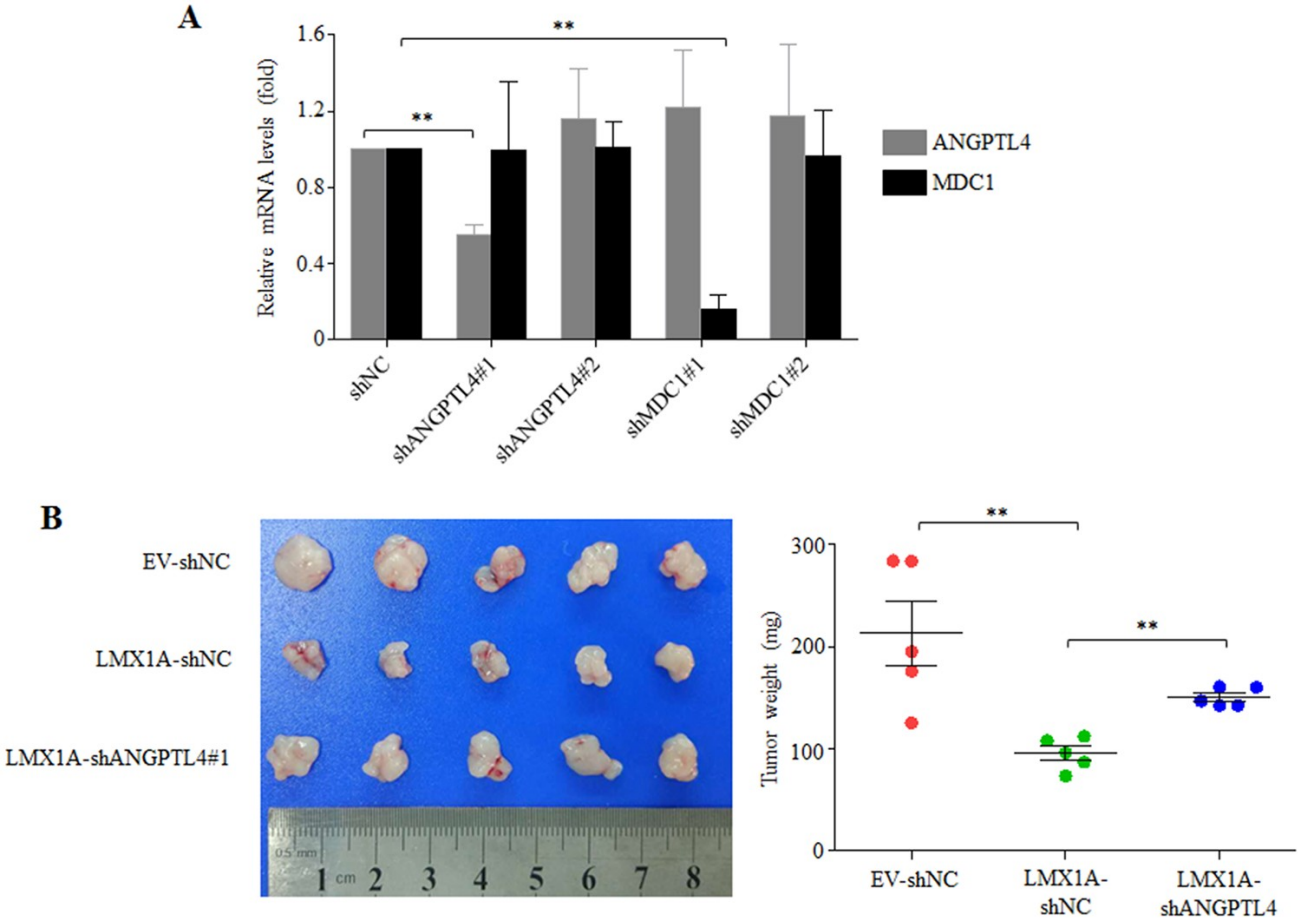
Knockdown of ANGPTL4 rescued the suppressive effect of LMX1A on tumorigenesis. (A) shRNAs specifically against ANGPTL4 (shANGPTL4#1 and shANGPTL4#2), MDC1 (shMDC1#1, shMDC1#2) and a scramble shRNA as negative control (shNC) were designed and the knockdown efficiencies of each shRNA were verified in AGS cells. Student’s t test. **, P<0.01. (B) AGS-EV cells infected with shNC virus (AGS-EV-shNC), and AGS-LMX1A cells infected with shNC (AGS-LMX1A-shNC) or shANGPTL4#1 (AGS-LMX1A-shANGPTL4#1) virus were subcutaneously injected into nude mice. Tumors were harvested 3 weeks later and weighed. Student’s t test. **, P<0.01.

Since LMX1A was proved to contribute to ANGPTL4 expression in gastric cancer cells, it was concerned whether up-regulation of ANGPTL4 could also influence the tumor suppression role of LMX1A. For this purpose, AGS cells with overexpression of LMX1A were subjected to knockdown of ANGPTL4 by shRNAs (shANGPTL4#1 and shANGPTL4#2), with shNC as negative control. As shown in Fig 4A, only shANGPTL4#1 effectively knocked down ANGPTL4 expression. Thus, we generated AGS-EV-shNC, AGS-LMX1A-shNC and AGS-LMX1A-shANGPTL4#1 cell lines, which were subcutaneously injected into nude mice. Three weeks after injection, tumors were harvested and weighed. The results showed that while LMX1A overexpression expectedly inhibited tumor growth, the inhibitory effect was reversed by knockdown of ANGPTL4 (Fig 4B). Taken together, our results indicate that the tumor suppressive role of LMX1A at least partly dependent on the up-regulation of ANGPTL4.

### LMX1A inhibits *C-Myc* protein expression

**Fig 5:**
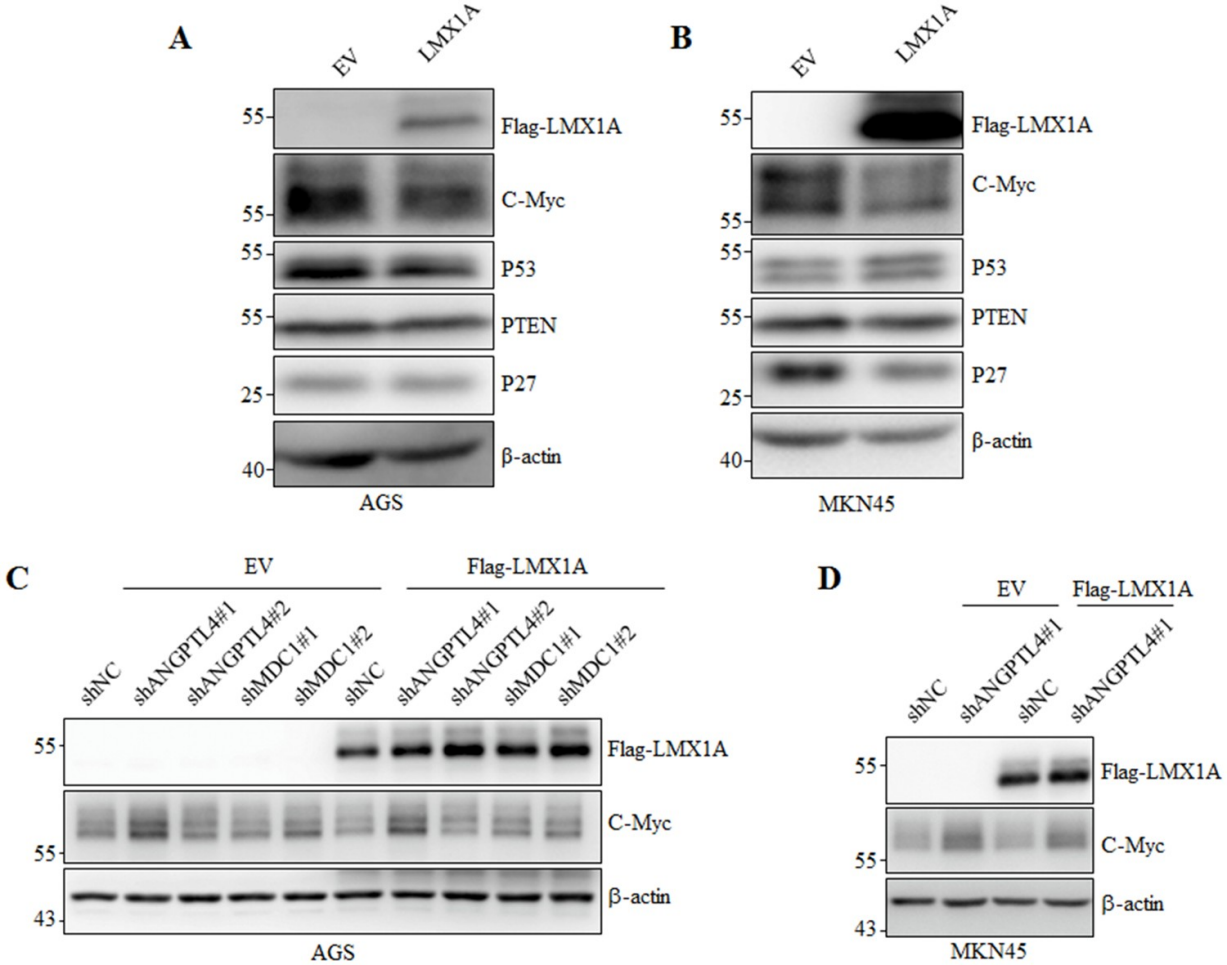
LMX1A inhibits the expression of oncogene C-Myc through ANGPTL4. (A, B) Immunoblotting of indicated proteins in AGS (A) and MKN45 (B) cells with or without LMX1A overexpression. (C) shRNAs against ANGPTL4 and MDC1 together with shNC were expressed in AGS-EV or AGS-LMX1A cells, and the indicated proteins were detected by immunoblotting. (D) shNC and shANGPTL4#1 were expressed in MKN45-EV or MKN45-LMX1A cells, and the indicated proteins were detected by immunoblotting.

RNA-sequencing detects regulatory gene relationships on mRNA level, but not regulations on protein level. We also detected the effects of LMX1A overexpression on several well-known oncogenes and tumor suppressors, including *C-Myc*, P53, PTEN and P27. Surprisingly, immunoblotting with specific antibodies against these proteins showed that LMX1A overexpression in both AGS and MKN45 cells significantly inhibited the expression of *C-Myc*, while P53, PTEN and P27 did not show significant change (Fig 5A and 5B). However, the *C-Myc* expression on RNA level was not regulated by LMX1A as shown in our RNA-sequencing data.

### ANGPTL4 mediates the inhibition of *C-Myc* by LMX1A

*C-Myc* is well known as one of the most potent drivers of tumorigenesis. The results showed that LMX1A inhibits *C-Myc* expression and regulates tumorigenesis through ANGPTL4, which promoted us to ask whether LMX1A regulated *C-Myc* through ANGPTL4. To verify this notion, shANGPTL4#1 and shANGPTL4#2 were transfected into AGS-EV or AGS-LMX1A cells. Surprisingly, knockdown of ANGPTL4 by shANGPTL4# 1 which efficiently knocked down ANGPTL4 expression (Fig 3A), significantly increased *C-Myc* level, both in AGS-EV or AGS-LMX1A cells to the similar degree, while shANGPTL4#2 which failed to knock down ANGPTL4 had no effect (Figs 3A and 5C). We also performed the same experiments with shRNAs against MDC1 (shMDC1#1 and MDC1#2) as negative controls, the results showed that although shMDC1#1 efficiently knocked down MDC1 expression (Fig 3A), both shMDC1#1 and MDC1#2 failed to affect *C-Myc* expression. The impact of ANGPTL4 on LMX1A-regulated *C-Myc* expression was recapitulated in MKN45 cells (Fig 5D). Taken together, our results clearly show that ANGPTL4 knockdown inversed the repression of *C-Myc* by LMX1A, indicating that LMX1A regulates *C-Myc* through up-regulating ANGPTL4.

## Discussion

Although the tumor suppressor role of LMX1A has long been proposed by us as well as others[4, 7], the underlying mechanism remain unrevealed. Our study for the first time aimed to find such clues. Considering LMX1A as a transcription factor, we used RNA sequencing to identify genes regulated by LMX1A overexpression, especially those who were positively and significantly regulated. Among the 13 genes that fit this profile, we chose 2 genes, MDC1 and ANGPTL4, which had been reported to act as tumor suppressors, to explain the function of LMX1A in this study. However, we could not rule out the possibility that other genes might also play important tumor suppressive roles, which should be interesting to be further validated in our future studies.

As only ANGPTL4 was verified to be consistently up-regulated by LMX1A in two gastric cancer cell lines, we chose ANGPTL4 for further functional analysis. Indeed, ANGPTL4, but not six other ANGPTL family members, showed co-expression with LMX1A in gastric cancer samples. What’s more, knockdown of ANGPTL4 reversed the inhibitory effect of LMX1A on tumorigenesis. Although there was great possibility that other pathways might exist, this is the first mechanistic explanation for the tumor suppressive role of LMX1A.

The LMX1A-ANGPTL4-*C-Myc* axis is proposed to be one of the mechanisms that explains the tumor suppressive role of LMX1A. In the experiment, we found that both LMX1A and ANGPTL4 are negative regulators of *C-Myc* protein, and LMX1A exerted its role through ANGPTL4. The important role of *C-Myc* as an oncogene is well established in cell growth and proliferation in gastric cancer[13]. An abnormal upregulation of *C-Myc* activated cell cycle and increased the cell proliferation of tumor cells, which in normal situation will be terminated and controlled by cell autophagy. More over, such cell autophagy disruption and *C-myc* oncogene overexpression in gastric cancer was reported to associate with a transformation of epithelial cells into adenocarcinoma and diffuse-type adenocarcinoma.[14] What’s more, the role of ANGPTL4 as tumor suppressor has also been reported.[12] It is also noticed the RNA level of *C-myc* have no change as an up-regulation of LMX1A in AGS cell line. It is proposed that the mRNA of *C-myc* is very few and unstable as transcriptional factor role of *C-Myc* protein. Generally. We thought that LMX1A increased the level of ANGPTL4, thus depress the expression of *C-myc* and reduce the cell proliferation of cancer cell. And In gastric cancer cells, such control mechanism was disrupted, the cancer cell increase the proliferation ability due to a uncontrolled overexpression of *C-Myc* and a lack of LMX1A expression.

## Supporting information

S1 File Quality control analysis of RNA-Seq. (ZIP)

S1 Table A list of potential targets for LMX1A in the promoter of Angptl4. (PNG)
