# Supplementary material for: LMX1A inhibits C-Myc expression through ANGPTL4 to exert tumor suppressive role in gastric cancer
Source: PLoS One. 2019 Sep 26;14(9):e0221640. doi: 10.1371/journal.pone.0221640 (PMC6762061; doi:10.1371/journal.pone.0221640)
Supplement: S1 File — (ZIP) [file pone.0221640.s001.zip › B_S358_L003_R1_001_fastqc.html]

B\_S358\_L003\_R1\_001.fastq.gz FastQC Report 

FastQC Report

���ڶ� 7 ���� 2019  
B\_S358\_L003\_R1\_001.fastq.gz

## Summary

- Basic Statistics
- Per base sequence quality
- Per tile sequence quality
- Per sequence quality scores
- Per base sequence content
- Per sequence GC content
- Per base N content
- Sequence Length Distribution
- Sequence Duplication Levels
- Overrepresented sequences
- Adapter Content

## Basic Statistics

| Measure | Value |
| --- | --- |
| Filename | B\_S358\_L003\_R1\_001.fastq.gz |
| File type | Conventional base calls |
| Encoding | Sanger / Illumina 1.9 |
| Total Sequences | 29525557 |
| Sequences flagged as poor quality | 0 |
| Sequence length | 150 |
| %GC | 51 |

## Per base sequence quality

## Per tile sequence quality

## Per sequence quality scores

## Per base sequence content

## Per sequence GC content

## Per base N content

## Sequence Length Distribution

## Sequence Duplication Levels

## Overrepresented sequences

No overrepresented sequences

## Adapter Content

Produced by FastQC (version 0.11.8)
