# Supplementary figures and images for: LMX1A inhibits C-Myc expression through ANGPTL4 to exert tumor suppressive role in gastric cancer
Source: PLoS One. 2019 Sep 26;14(9):e0221640. doi: 10.1371/journal.pone.0221640 (PMC6762061; doi:10.1371/journal.pone.0221640)

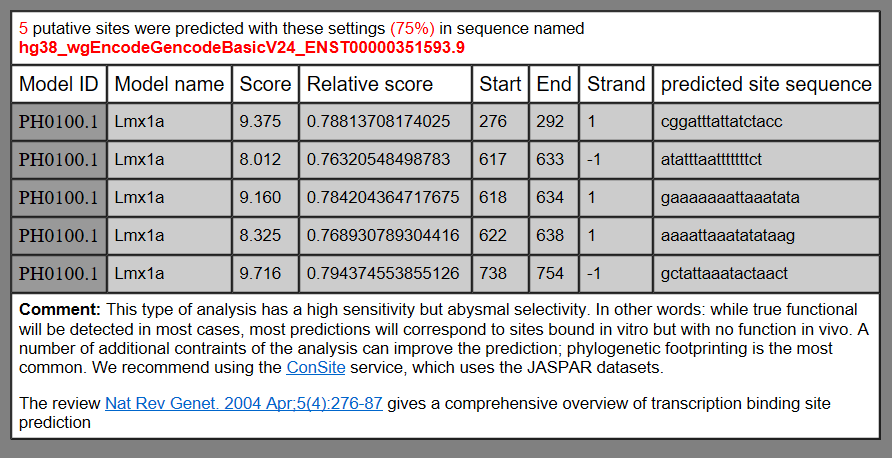

Supplement: S1 Table — (PNG) [file pone.0221640.s002.png]
